# Incidental Diagnosis of Osteopoikilosis Using Plain Radiography in a Resource-Limited Tier 2 City: A Case Report and Brief Literature Review

**DOI:** 10.7759/cureus.110153

**Published:** 2026-06-02

**Authors:** Rasim K Yoosaf, Mohamed Afsal C H., Sudev Raghunathan

**Affiliations:** 1 Orthopaedics and Trauma, Kerala Medical College, Mangode, Palakkad, IND; 2 Orthopaedics, Kerala Medical College, Mangode, Palakkad, IND

**Keywords:** bony lesions in knee, differentials for bony lesions, enostoses, knee pain, lemd 3, management of osteopoikilosis, orthopaedics, osteopoikilosis, tier 2

## Abstract

Osteopoikilosis is a rare, benign condition affecting bone development, characterized by many small areas of dense bone (enostoses) that are usually found unexpectedly during X-rays. Because its appearance on X-ray can resemble cancer spread to the bone (osteoblastic metastases), diagnosis can be difficult, particularly in areas where more sophisticated and expensive scans are not easily available. We present the case of a 26-year-old woman from an economically constrained background, with mild knee joint pain, who was diagnosed using standard X-rays alone. The typical pattern on the X-ray allowed a confident diagnosis of osteopoikilosis and ruled out more serious illnesses. This highlights the value of recognizing the specific features seen on imaging to prevent unnecessary tests, costs, and anxiety for the patient. A review of published studies is also included to emphasize how to make a diagnosis when resources are limited.

## Introduction

Osteopoikilosis, an infrequent and benign bone-hardening condition, is defined by multiple small, symmetrical, round or oval areas of increased bone density, commonly called bone islands or enostoses [1,2]. Albers-Schönberg first described it in 1915, and it’s frequently passed down within families in a dominant inheritance pattern and has been linked to changes in the LEMD3 gene [3]. It’s thought to affect about 1 in 50,000 people, but this number might be lower because the majority of the population with the condition have no symptoms demonstrable [4,5]. It is most often discovered by chance during an X-ray taken for an unrelated issue [6].

The main importance of diagnosing osteopoikilosis is to tell it apart from more problematic conditions like osteoblastic metastases, mastocytosis, and tuberous sclerosis [7,8]. This is especially important where access to more advanced scans like computed tomography (CT) scans, magnetic resonance imaging (MRI), or bone scans is restricted. Incorrectly interpreting the condition could lead to unnecessary referrals to specialists, more invasive procedures, and financial difficulties.

This case report demonstrates the usefulness of standard X-rays and careful assessment of the patient’s situation in diagnosing osteopoikilosis in a setting with limited resources in a tier 2 city (population size between 50,000 and 99,000) in India.

## Case presentation

A 26-year-old woman came to the outpatient clinic complaining of a dull, aching pain in both knees, which had occurred on and off for a year. The pain wasn’t severe, didn’t travel elsewhere, and wasn’t accompanied by swelling, redness, or stiffness in the morning. She did not experience any pain in any other joints. She had not injured her knees, hadn’t had a fever, lost weight, or experienced pain at night. The patient works as a domestic helper and comes from a low-income family; she said she would find it difficult to pay for more detailed investigations. She has no known history of cancer or long-term illness, and her family history doesn’t provide any relevant details. When examined, her vital signs were stable. A general examination showed no paleness, swollen lymph nodes, or wasting of muscle. Her musculoskeletal examination revealed no swelling or distortion of the joints, full movement in all joints, and no tenderness in a specific area. A systemic examination was unremarkable. Because of the patient’s financial situation, tests were limited to basic blood tests and X-rays. Her complete blood count was within the normal range. Her erythrocyte sedimentation rate (ESR) was normal. Her calcium, phosphate, and alkaline phosphatase levels in her blood were all normal.

X-rays were taken of both knees from the front, side, and with the knee bent (anteroposterior, lateral, and skyline views). The X-rays showed several small (2-10 mm), clearly defined, uniformly dense areas of hardening of the bone. These areas were arranged in a symmetrical pattern. They were mostly near the joints (at the ends of the bones - epiphysis and metaphysis). There was no breakdown of the outer layer of the bone (cortex), thickening of the outer layer of the bone, or abnormality of the surrounding soft tissues.

**Figure 1:**
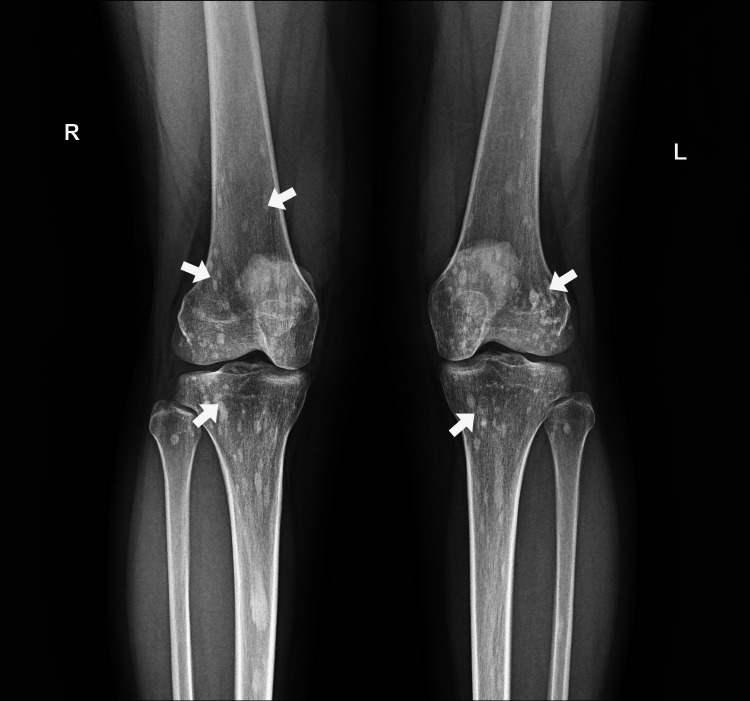
X-ray AP view of bilateral knee joint showing multiple lesions White arrows show multiple lesions in the bilateral knees in a symmetric pattern; the small, well-defined, ovoid/sclerotic nature of the lesions shows periarticular distribution. AP: anteroposterior; R: right lower limb; L: left lower limb

**Figure 2:**
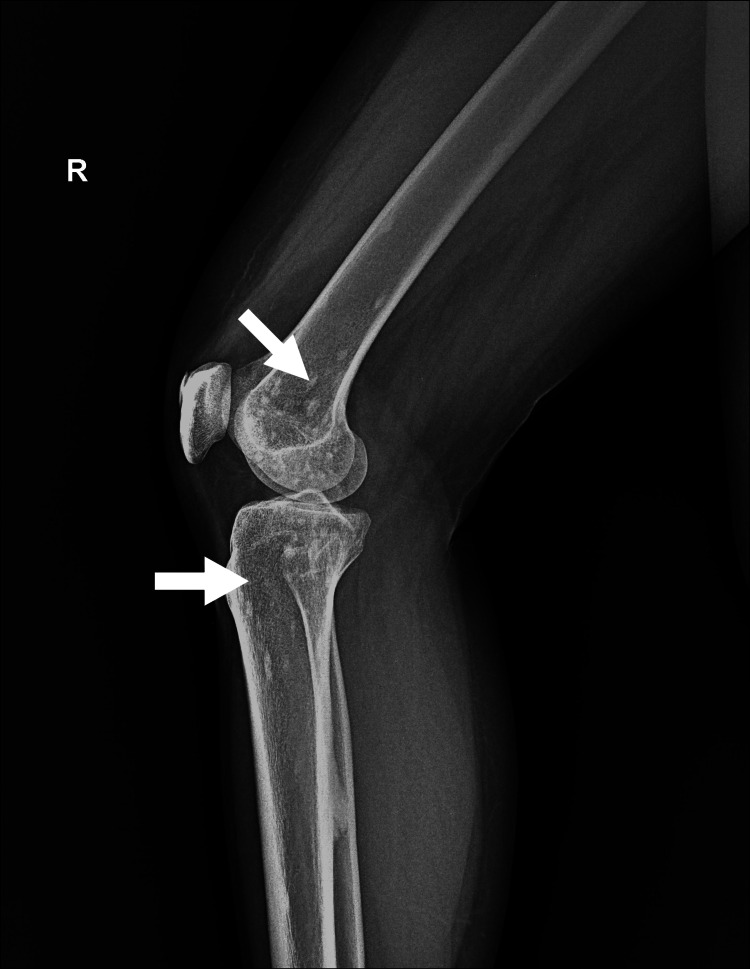
X-ray lateral view of the right knee White arrows represent the multiple bony lesions in both the femur and tibia. R: right lower limb

**Figure 3:**
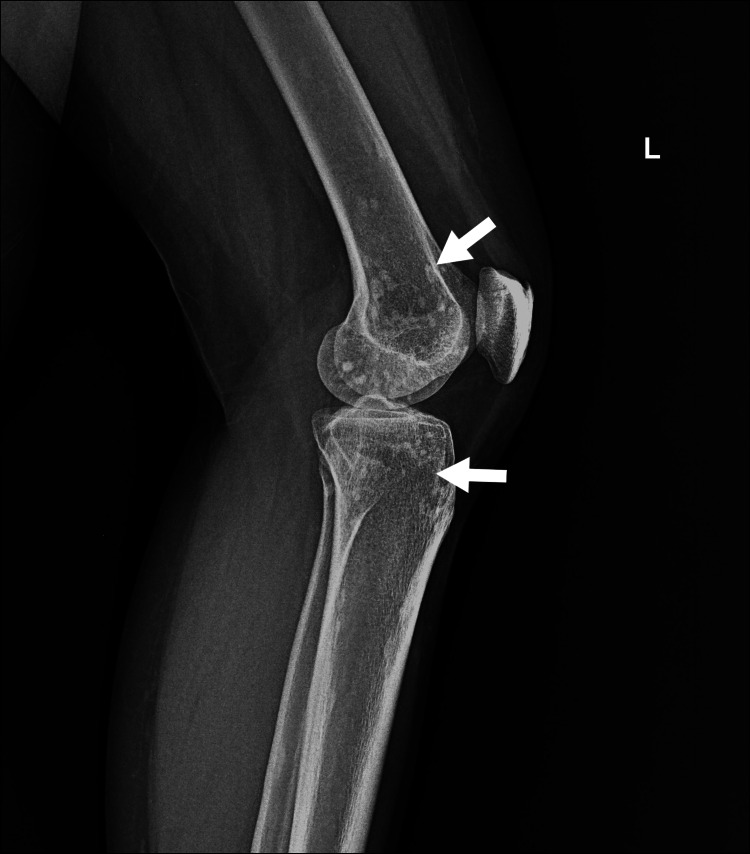
X-ray lateral view of the left knee joint White arrows show the multiple bony lesions in both the femur and tibia, which are symmetrical on the other side. L: left lower limb

**Figure 4:**
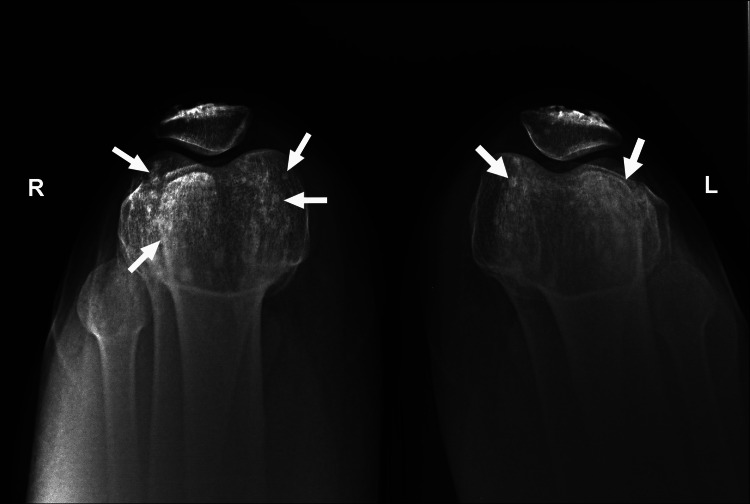
X-ray skyline view of the bilateral knee joint White arrows mark the bony lesions. A characteristic finding noted here is that there is no break in the cortex of the involved bones, including the patella. R: right lower limb; L: left lower limb

Based on these findings, as shown in Figures 1-4, a diagnosis of osteopoikilosis was made.

Based on the above mentioned clinical symptoms and X-ray findings, the following differential diagnosis were considered with osteoblastic metastases being the most prominent one, but was ruled out as the patient didn't present with the typical features including multiple bony lesions, which are typically not symmetrical, vary in size, can cause destruction of the outer layer of the bone and thickening around the bone, and are usually associated with general symptoms of illness [7,8]. The other differential diagnosis that were considered in the absence of a bone biopsy as the patient was not willing for a invasive procedure was mastocytosis, which usually show both areas of bone breakdown and hardening, and is often linked to skin problems [2]; tuberous sclerosis, which is linked to areas of hardening within the bone (cortical tubers), seizures and skin issues [9]; melorheostosis, which has a particular "flowing candle wax" pattern along the outer layer of the bone [1]; and Paget's disease, which causes the bone to expand and the outer layer to become thicker [9]. The lack of general illness, the consistent appearance of the lesions, and their symmetrical location around the joints all pointed towards osteopoikilosis.

In a more comprehensive setting, additional tests like a bone scan or MRI could be used to confirm the diagnosis. However, in this instance, there was no access to a bone scan, and the patient was not affordable for an MRI, so the diagnosis was made by recognizing the typical pattern on the X-ray as seen in the above-mentioned figures [2,6]. This avoided placing an unnecessary financial strain on the patient. The patient was counselled about the condition, given reassurance, and was started on oral analgesics and knee physiotherapy exercises, which included quadriceps and hamstring strengthening exercises. The patient was followed up after two weeks and reported marked improvement of symptoms. The patient was then followed up after six weeks, following which the patient resumed regular work after attaining satisfactory pain relief.

## Discussion

Osteopoikilosis falls within the spectrum of sclerosing bone dysplasias and typically doesn't cause any symptoms [1,10]. If symptoms are present, they are generally mild and not specifically related to osteopoikilosis, such as aches or stiffness in the joints [4]. The development of the condition has a basis in mutations of the LEMD3 gene; this impacts bone morphogenetic protein (BMP) signalling, and consequently causes the bone to form abnormally [3]. Osteopoikilosis is most noticeably characterized by multiple enostoses, a symmetrical arrangement of these bony growths, a tendency to occur around joints, and a consistent shape and opacity [1,2]. The bones most frequently affected are the pelvis, the bones of the wrists and ankles (carpals and tarsals), and the ends of the longer bones - the tibia, femur, and others [6].

A crucial part of diagnosis is differentiating osteopoikilosis from osteoblastic metastases. The key points of contrast are that osteopoikilosis has a symmetrical distribution, lesions of a uniform size, commonly causes no symptoms, and does not show on a bone scan [7,8]. Metastases, on the other hand, are asymmetrical, have differing lesion sizes, usually cause symptoms, and are indicated by increased uptake on a bone scan [8]. In countries with good medical resources, a bone scan will not show increased uptake with osteopoikilosis, and this finding supports the diagnosis [7]. Unfortunately, these scans are often unavailable in poorer countries and cities. Within economically disadvantaged groups, the possibility of excessive and expensive testing exists; this can lead to financial ruin for the patient. Incorrect diagnosis can lead to emotional problems, and specialist medical care is difficult to obtain. Therefore, it is important to use affordable methods like a standard X-ray. Osteopoikilosis can occur alongside Buschke-Ollendorff syndrome (a condition involving connective tissue skin growths - small, multiple, benign skin papules/nodules), and, infrequently, unusual abnormalities of the skeleton [2]. However, none of these were present in this particular patient.

## Conclusions

Osteopoikilosis is a benign sclerosing dysplasia, which can be accurately diagnosed through plain radiography if the typical characteristics are visible. In areas where medical resources are limited, careful consideration of the patient’s condition and the X-ray results is enough to rule out serious conditions like cancer spreading to the bone. Doctors need to know about osteopoikilosis in order to avoid wrong diagnoses, unnecessary tests, and the financial hardship these can cause patients.
